# (*E*)-Methyl 2,6-dichloro-*N*-cyano­benzimidate

**DOI:** 10.1107/S1600536809026452

**Published:** 2009-07-11

**Authors:** Xiao-Ai Wu, Ping Yin, Ling He, Zi-Cheng Li, Wen-Cai Huang

**Affiliations:** a Key Laboratory of Drug Targeting and Drug-Delivery Systems of the Ministry of Education, Department of Medicinal Chemistry, West China School of Pharmacy, Sichuan University, Chengdu, 610041, People’s Republic of China; b Department of Pharmaceutical and Bioengineering, School of Chemical Engineering, Sichuan University, Chengdu 610065, People’s Republic of China

## Abstract

The mol­ecule of the title compound, C_9_H_6_Cl_2_N_2_O, displays an *E* conformation about the C=N double bond. The *N*-cyano­imidate fragment is substanti­ally planar [maximum deviation 0.010 (4) Å] and perpendicular to the benzene ring [dihedral angle = 88.50 (14)°]. In the crystal packing, inter­molecular Cl⋯Cl inter­actions [3.490 (2) Å] are observed.

## Related literature

For the synthesis of substituted cyano­imidates, see: Huffman & Schaefer (1963 ▶). For the crystal structures of compounds containing the *N*-cyano­imidate fragment, see: Zöllinger *et al.* (2006 ▶); Ponomareva *et al.* (1995 ▶); Jäger *et al.* (1996 ▶). For details of halogen⋯halogen inter­actions, see: Desiraju & Parthasarathy (1989 ▶).
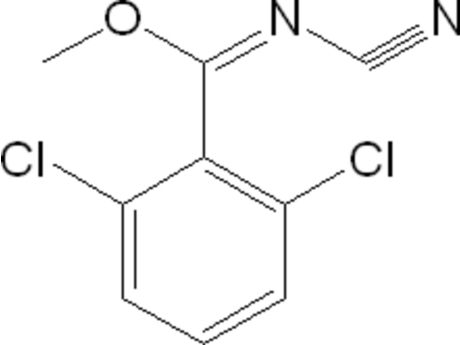


## Experimental

### 

#### Crystal data

C_9_H_6_Cl_2_N_2_O
*M* *_r_* = 229.06
Monoclinic, 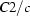

*a* = 21.199 (4) Å
*b* = 8.548 (3) Å
*c* = 15.005 (4) Å
β = 128.49 (4)°
*V* = 2128.2 (16) Å^3^
*Z* = 8
Mo *K*α radiation
μ = 0.58 mm^−1^
*T* = 291 K
0.52 × 0.46 × 0.28 mm

#### Data collection

Enraf–Nonius CAD-4 diffractometer
Absorption correction: spherical (*WinGX*; Farrugia, 1999 ▶) *T*_min_ = 0.754, *T*_max_ = 0.855
2116 measured reflections
1948 independent reflections
1167 reflections with *I* > 2σ(*I*)
*R*_int_ = 0.016
3 standard reflections every 120 reflections intensity decay: 3.8%

#### Refinement

*R*[*F* ^2^ > 2σ(*F* ^2^)] = 0.054
*wR*(*F* ^2^) = 0.170
*S* = 1.04
1948 reflections
129 parameters
H-atom parameters constrained
Δρ_max_ = 0.30 e Å^−3^
Δρ_min_ = −0.32 e Å^−3^

### 

Data collection: *DIFRAC* (Gabe & White, 1993 ▶); cell refinement: *DIFRAC*; data reduction: *NRCVAX* (Gabe *et al.*, 1989 ▶); program(s) used to solve structure: *SHELXS97* (Sheldrick, 2008 ▶); program(s) used to refine structure: *SHELXL97* (Sheldrick, 2008 ▶); molecular graphics: *ORTEP-3 for Windows* (Farrugia, 1997 ▶); software used to prepare material for publication: *SHELXL97*.

## Supplementary Material

Crystal structure: contains datablocks global, I. DOI: 10.1107/S1600536809026452/rz2343sup1.cif

Structure factors: contains datablocks I. DOI: 10.1107/S1600536809026452/rz2343Isup2.hkl

Additional supplementary materials:  crystallographic information; 3D view; checkCIF report

## Figures and Tables

**Fig. 1.**
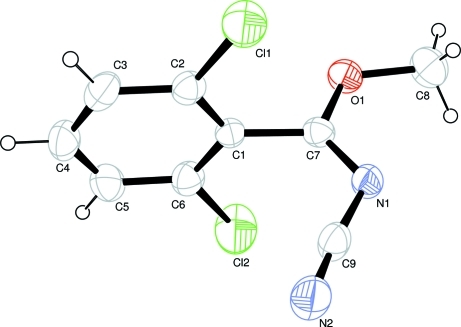
The molecular structure of the title compound, with displacement ellipsoids drawn at the 50% probability level.

**Fig. 2.**
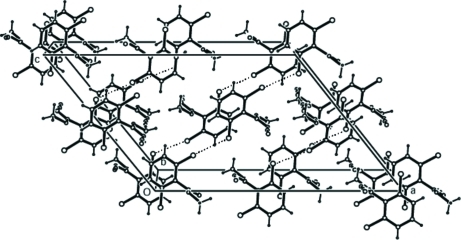
Crystal packing of the title compound approximately viewed along the b axis. Cl···Cl interactions are shown as dotted lines.

## supplementary crystallographic information

### Comment

In the course of our studies aimed to prepare substituted cyanoimidates (Huffman & Schaefer, 1963) from the corresponding aromatic aldehyde by oxidation using 1-bromopyrrolidine-2,5-dione, the title compound was obtained in 84% yield, and its crystal structure is reported herein.

The molecule of the title compound (Fig. 1) displays an *E* conformation about the C═N double bond. The O—C═N—C≡N *N*-cyanoimidate fragment is substantially planar [maximum deviation 0.010 (4) Å for atom N2] and forms a dihedral angle of 88.50 (14)° with the plane of the benzene ring. As far as the authors are aware, crystal structures reporting the presence of the *N*-cyanoimidate fragment are very rare, the only examples found in the literature being 1-(2,3-dibromo-10-oxo-7,8-dihydro-6H,10H-dipyrrolo[1,2-a:1',2'-d]pyrazin-5-yl)-2-(2,6-dimethylphenyl)-3-cyanoisourea monohydrate (Zöllinger *et al.*, 2006), *catena*-poly-[(µ^6^-benzoylcyanamido)-thallium(I)] (Ponomareva *et al.*, 1995) and tris(ethylenediamine)-nickel(II) bis[2-methyl-4-chlorophenoxy(cyanamido)acetate] (Jäger *et al.*, 1996). In the crystal structure, molecules are connected by intermolecular Cl···Cl interactions of 3.490 (2) Å (Desiraju & Parthasarathy, 1989) into one-dimensional chains running parallel to [101].

### Experimental

A mixture of 2,6-dichlorobenzaldehyde (1 mmol), H_2_NCN (3 equiv) and t-BuONa (3 equiv) in MeOH (8 ml) was stirred for 30 min at room temperature, then N-bromosuccinimide (NBS; 3 equiv) was added. After stirring for 12 h at 323 K, the mixture was purified by flash chromatography on silica gel with petroleum ether/ethyl acetate (100:1–25:1 *v*/*v*) as eluent to give the title compound in 84% yield. Colourless crystals suitable for X-ray analysis were obtained by slow evaporation of a petroleum ether/acetyl acetate solution (25:1 *v*/*v*) at room temperature.

### Refinement

H atoms were positioned geometrically (C—H = 0.93–0.96 Å) and refined using a riding model, with *U*_iso_(H) = 1.2*U*_eq_(C) or 1.5*U*_eq_(C) for methyl H atoms.

### Crystal data

**Table:**

C_9_H_6_Cl_2_N_2_O	*F*(000) = 928
*M**_r_* = 229.06	*D*_x_ = 1.430 Mg m^−^^3^
Monoclinic, *C*2/*c*	Mo *K*α radiation, λ = 0.71073 Å
Hall symbol: -C 2yc	Cell parameters from 28 reflections
*a* = 21.199 (4) Å	θ = 4.8–9.2°
*b* = 8.548 (3) Å	µ = 0.58 mm^−^^1^
*c* = 15.005 (4) Å	*T* = 291 K
β = 128.49 (4)°	Block, colourless
*V* = 2128.2 (16) Å^3^	0.52 × 0.46 × 0.28 mm
*Z* = 8	

### Data collection

**Table:**

Enraf–Nonius CAD-4 diffractometer	1167 reflections with *I* > 2σ(*I*)
Radiation source: fine-focus sealed tube	*R*_int_ = 0.016
graphite	θ_max_ = 25.5°, θ_min_ = 2.5°
ω/2θ scans	*h* = −25→17
Absorption correction: for a sphere (*WinGX*; Farrugia, 1999)	*k* = 0→10
*T*_min_ = 0.754, *T*_max_ = 0.855	*l* = −18→18
2116 measured reflections	3 standard reflections every 120 reflections
1948 independent reflections	intensity decay: 3.8%

### Refinement

**Table:**

Refinement on *F*^2^	Secondary atom site location: difference Fourier map
Least-squares matrix: full	Hydrogen site location: inferred from neighbouring sites
*R*[*F*^2^ > 2σ(*F*^2^)] = 0.054	H-atom parameters constrained
*wR*(*F*^2^) = 0.170	*w* = 1/[σ^2^(*F*_o_^2^) + (0.1053*P*)^2^] where *P* = (*F*_o_^2^ + 2*F*_c_^2^)/3
*S* = 1.03	(Δ/σ)_max_ < 0.001
1948 reflections	Δρ_max_ = 0.30 e Å^−^^3^
129 parameters	Δρ_min_ = −0.32 e Å^−^^3^
0 restraints	Extinction correction: *SHELXL97* (Sheldrick, 2008), Fc^*^=kFc[1+0.001xFc^2^λ^3^/sin(2θ)]^-1/4^
Primary atom site location: structure-invariant direct methods	Extinction coefficient: 0.0069 (15)

### Special details

**Table:**

Geometry. All e.s.d.'s (except the e.s.d. in the dihedral angle between two l.s. planes) are estimated using the full covariance matrix. The cell e.s.d.'s are taken into account individually in the estimation of e.s.d.'s in distances, angles and torsion angles; correlations between e.s.d.'s in cell parameters are only used when they are defined by crystal symmetry. An approximate (isotropic) treatment of cell e.s.d.'s is used for estimating e.s.d.'s involving l.s. planes.
Refinement. Refinement of *F*^2^ against ALL reflections. The weighted *R*-factor *wR* and goodness of fit *S* are based on *F*^2^, conventional *R*-factors *R* are based on *F*, with *F* set to zero for negative *F*^2^. The threshold expression of *F*^2^ > σ(*F*^2^) is used only for calculating *R*-factors(gt) *etc*. and is not relevant to the choice of reflections for refinement. *R*-factors based on *F*^2^ are statistically about twice as large as those based on *F*, and *R*- factors based on ALL data will be even larger.

### Fractional atomic coordinates and isotropic or equivalent isotropic displacement parameters (Å^2^)

**Table:**

	*x*	*y*	*z*	*U*_iso_*/*U*_eq_	
Cl1	0.54862 (5)	0.67130 (14)	0.65350 (8)	0.0881 (5)	
Cl2	0.24528 (6)	0.72469 (18)	0.26898 (9)	0.1121 (6)	
O1	0.36902 (14)	0.4760 (2)	0.5066 (2)	0.0672 (7)	
N1	0.35148 (15)	0.7020 (3)	0.5663 (2)	0.0563 (7)	
N2	0.3600 (2)	0.9913 (4)	0.5784 (3)	0.0866 (10)	
C1	0.40050 (16)	0.7037 (3)	0.4547 (2)	0.0472 (7)	
C2	0.48117 (18)	0.7300 (3)	0.5135 (3)	0.0545 (8)	
C3	0.5094 (2)	0.8077 (4)	0.4632 (3)	0.0675 (9)	
H3	0.5642	0.8259	0.5039	0.081*	
C4	0.4550 (3)	0.8571 (4)	0.3523 (3)	0.0745 (10)	
H4	0.4735	0.9086	0.3179	0.089*	
C5	0.3742 (2)	0.8318 (4)	0.2917 (3)	0.0750 (10)	
H5	0.3379	0.8664	0.2169	0.090*	
C6	0.3471 (2)	0.7542 (4)	0.3432 (3)	0.0613 (9)	
C7	0.37157 (16)	0.6295 (3)	0.5129 (2)	0.0486 (7)	
C8	0.3474 (3)	0.3932 (4)	0.5685 (4)	0.0930 (13)	
H8A	0.3881	0.4102	0.6487	0.139*	
H8B	0.3433	0.2833	0.5525	0.139*	
H8C	0.2966	0.4312	0.5453	0.139*	
C9	0.3568 (2)	0.8581 (4)	0.5699 (3)	0.0619 (8)	

### Atomic displacement parameters (Å^2^)

**Table:**

	*U*^11^	*U*^22^	*U*^33^	*U*^12^	*U*^13^	*U*^23^
Cl1	0.0583 (5)	0.1217 (9)	0.0646 (6)	0.0019 (5)	0.0285 (5)	0.0127 (5)
Cl2	0.0589 (6)	0.1721 (13)	0.0731 (7)	−0.0024 (6)	0.0252 (5)	0.0231 (7)
O1	0.0903 (16)	0.0367 (12)	0.0958 (17)	0.0011 (10)	0.0684 (15)	0.0009 (10)
N1	0.0733 (17)	0.0436 (14)	0.0707 (16)	−0.0010 (11)	0.0540 (15)	0.0000 (11)
N2	0.129 (3)	0.0544 (18)	0.122 (3)	−0.0121 (17)	0.100 (3)	−0.0177 (17)
C1	0.0566 (16)	0.0401 (14)	0.0550 (16)	0.0025 (12)	0.0397 (14)	−0.0008 (12)
C2	0.0587 (17)	0.0537 (17)	0.0574 (17)	0.0016 (13)	0.0393 (15)	−0.0039 (13)
C3	0.0639 (19)	0.070 (2)	0.087 (2)	−0.0075 (16)	0.056 (2)	−0.0115 (18)
C4	0.103 (3)	0.069 (2)	0.090 (3)	0.000 (2)	0.079 (2)	0.0017 (19)
C5	0.096 (3)	0.078 (2)	0.066 (2)	0.013 (2)	0.058 (2)	0.0132 (18)
C6	0.0620 (18)	0.068 (2)	0.0564 (18)	0.0022 (15)	0.0382 (16)	0.0003 (15)
C7	0.0502 (15)	0.0413 (15)	0.0535 (16)	0.0004 (12)	0.0319 (13)	−0.0001 (12)
C8	0.123 (3)	0.050 (2)	0.144 (4)	−0.004 (2)	0.101 (3)	0.014 (2)
C9	0.080 (2)	0.055 (2)	0.076 (2)	−0.0076 (16)	0.0612 (19)	−0.0101 (16)

### Geometric parameters (Å, °)

**Table:**

Cl1—C2	1.724 (3)	C2—C3	1.389 (4)
Cl2—C6	1.724 (4)	C3—C4	1.374 (5)
O1—C7	1.314 (3)	C3—H3	0.9300
O1—C8	1.451 (4)	C4—C5	1.367 (5)
N1—C7	1.278 (4)	C4—H4	0.9300
N1—C9	1.338 (4)	C5—C6	1.385 (5)
N2—C9	1.143 (4)	C5—H5	0.9300
C1—C2	1.370 (4)	C8—H8A	0.9600
C1—C6	1.382 (4)	C8—H8B	0.9600
C1—C7	1.486 (4)	C8—H8C	0.9600
			
C7—O1—C8	117.2 (3)	C4—C5—H5	120.4
C7—N1—C9	117.1 (3)	C6—C5—H5	120.4
C2—C1—C6	118.8 (3)	C1—C6—C5	120.9 (3)
C2—C1—C7	119.9 (3)	C1—C6—Cl2	119.2 (3)
C6—C1—C7	121.3 (3)	C5—C6—Cl2	119.8 (3)
C1—C2—C3	121.1 (3)	N1—C7—O1	121.0 (3)
C1—C2—Cl1	119.5 (2)	N1—C7—C1	125.6 (2)
C3—C2—Cl1	119.4 (2)	O1—C7—C1	113.4 (2)
C4—C3—C2	119.0 (3)	O1—C8—H8A	109.5
C4—C3—H3	120.5	O1—C8—H8B	109.5
C2—C3—H3	120.5	H8A—C8—H8B	109.5
C5—C4—C3	121.1 (3)	O1—C8—H8C	109.5
C5—C4—H4	119.5	H8A—C8—H8C	109.5
C3—C4—H4	119.5	H8B—C8—H8C	109.5
C4—C5—C6	119.2 (3)	N2—C9—N1	174.8 (4)
			
C6—C1—C2—C3	0.8 (4)	C7—C1—C6—Cl2	−2.1 (4)
C7—C1—C2—C3	−176.1 (3)	C4—C5—C6—C1	0.6 (5)
C6—C1—C2—Cl1	178.8 (2)	C4—C5—C6—Cl2	178.7 (3)
C7—C1—C2—Cl1	1.9 (4)	C9—N1—C7—O1	179.2 (3)
C1—C2—C3—C4	−0.6 (5)	C9—N1—C7—C1	0.2 (4)
Cl1—C2—C3—C4	−178.6 (3)	C8—O1—C7—N1	−3.7 (4)
C2—C3—C4—C5	0.4 (5)	C8—O1—C7—C1	175.4 (3)
C3—C4—C5—C6	−0.4 (5)	C2—C1—C7—N1	89.7 (4)
C2—C1—C6—C5	−0.8 (5)	C6—C1—C7—N1	−87.1 (4)
C7—C1—C6—C5	176.0 (3)	C2—C1—C7—O1	−89.4 (3)
C2—C1—C6—Cl2	−178.9 (2)	C6—C1—C7—O1	93.8 (3)
